# Supplementary material for: Dispensable players: N-WASP and WASP are not crucial for homology-directed DNA repair
Source: EMBO Rep. 2026 Apr 10;27(10):2798–822. doi: 10.1038/s44319-026-00771-y (PMC13219447; doi:10.1038/s44319-026-00771-y)
Supplement: Supplementary file 2 — Source data Fig. 1 [file 44319_2026_771_MOESM2_ESM.zip › Figure 1/1A/WB - 3 cell lines.pptx]

## Slide 1
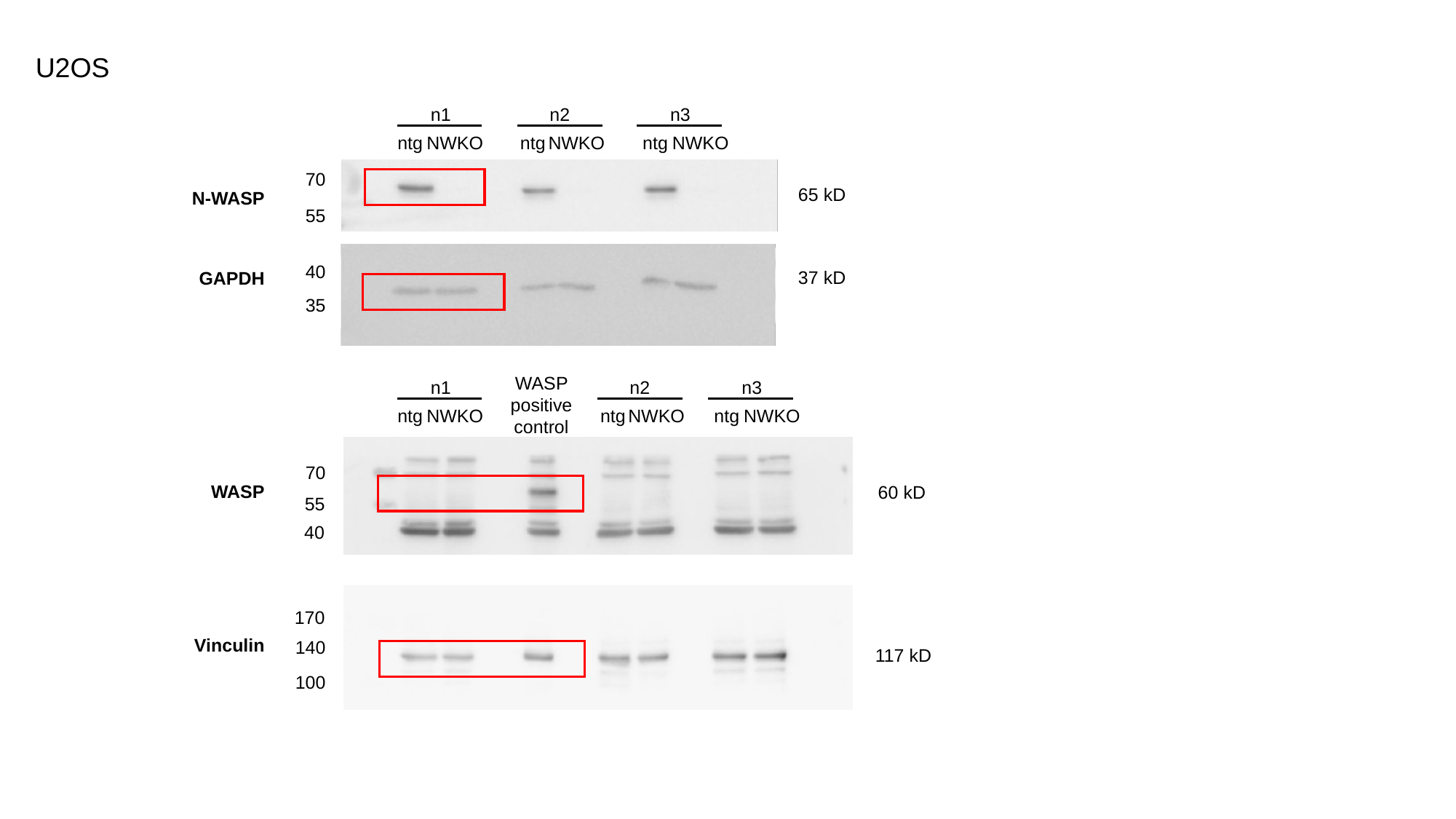

U2OS
n1
ntg
NWKO
n2
ntg
NWKO
n3
ntg
NWKO
70
65 kD
N-WASP
55
40
37 kD
GAPDH
35
WASP positive control
n1
ntg
NWKO
n2
ntg
NWKO
n3
ntg
NWKO
70
WASP
60 kD
55
40
170
Vinculin
140
117 kD
100

## Slide 2
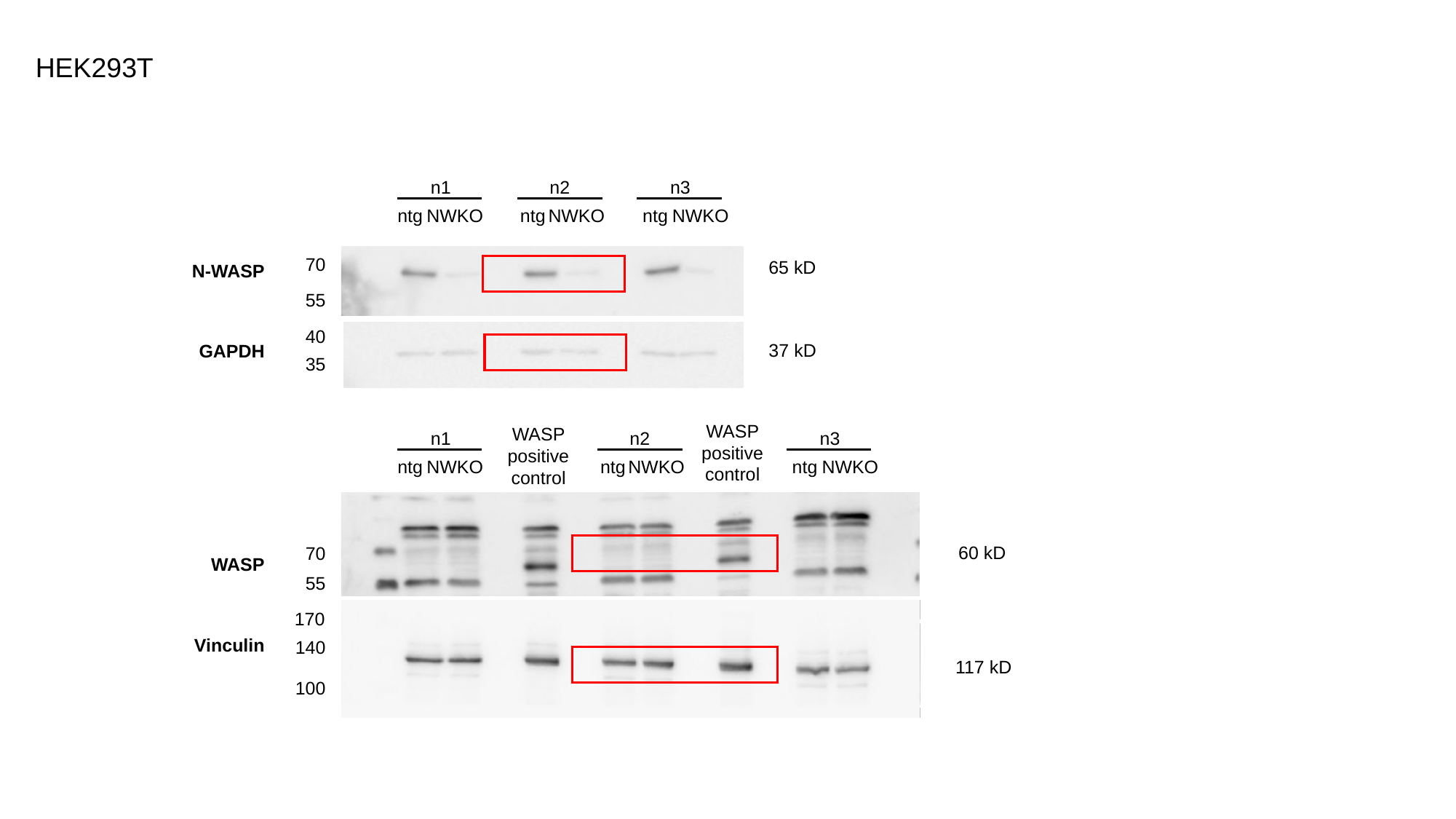

HEK293T
n1
ntg
NWKO
n2
ntg
NWKO
n3
ntg
NWKO
70
65 kD
N-WASP
55
40
37 kD
GAPDH
35
WASP positive control
WASP positive control
n1
ntg
NWKO
n2
ntg
NWKO
n3
ntg
NWKO
60 kD
70
WASP
55
170
Vinculin
140
117 kD
100

## Slide 3
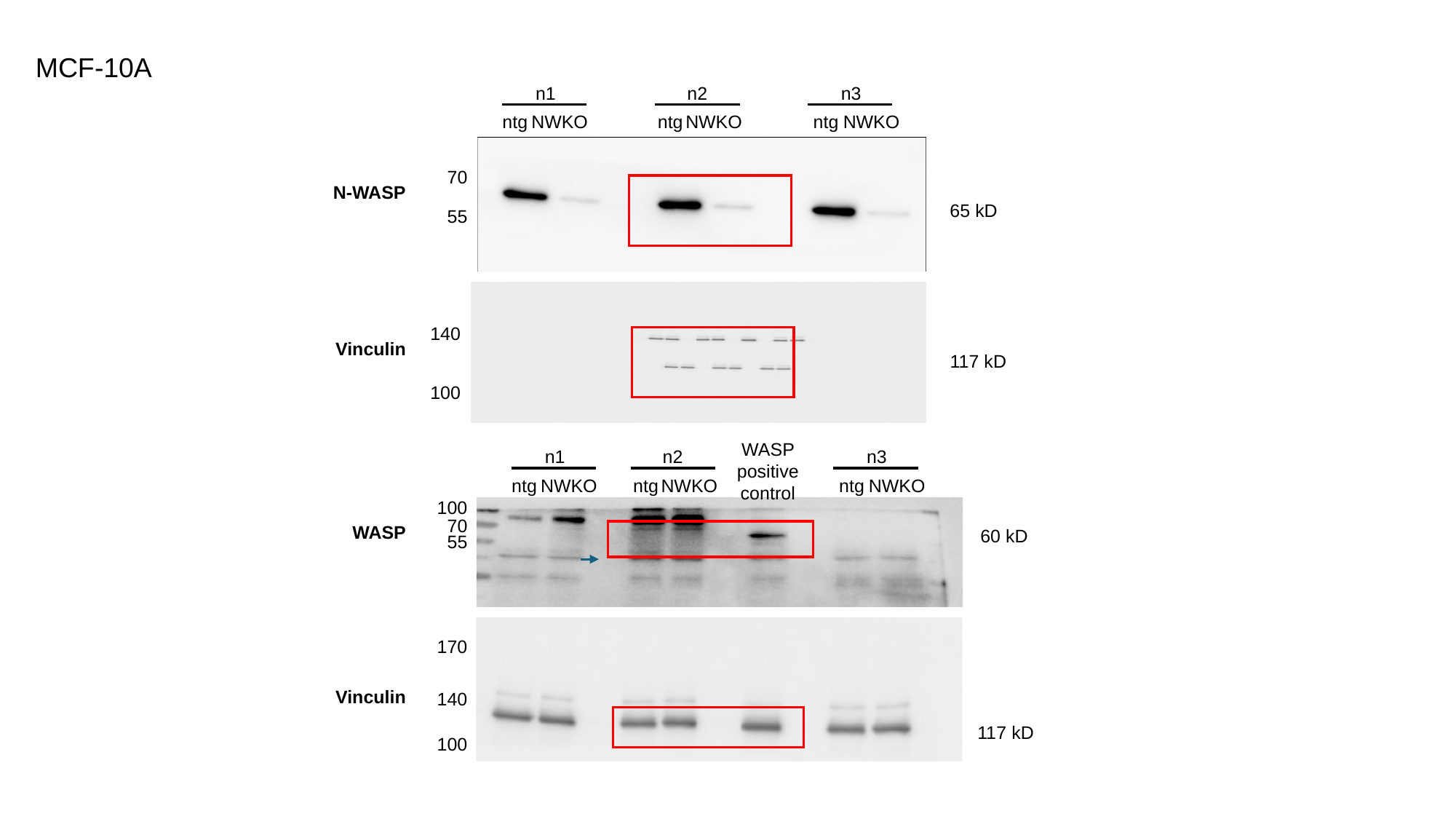

MCF-10A
n1
ntg
NWKO
n2
ntg
NWKO
n3
ntg
NWKO
70
N-WASP
65 kD
55
140
Vinculin
117 kD
100
WASP positive control
n1
ntg
NWKO
n2
ntg
NWKO
n3
ntg
NWKO
100
70
WASP
60 kD
55
170
140
100
Vinculin
117 kD
