# Supplementary material for: Dispensable players: N-WASP and WASP are not crucial for homology-directed DNA repair
Source: EMBO Rep. 2026 Apr 10;27(10):2798–822. doi: 10.1038/s44319-026-00771-y (PMC13219447; doi:10.1038/s44319-026-00771-y)
Supplement: Supplementary file 3 — Source data Fig. 4 [file 44319_2026_771_MOESM3_ESM.zip › Figure 4/4B/Western Blot.pptx]

## Slide 1
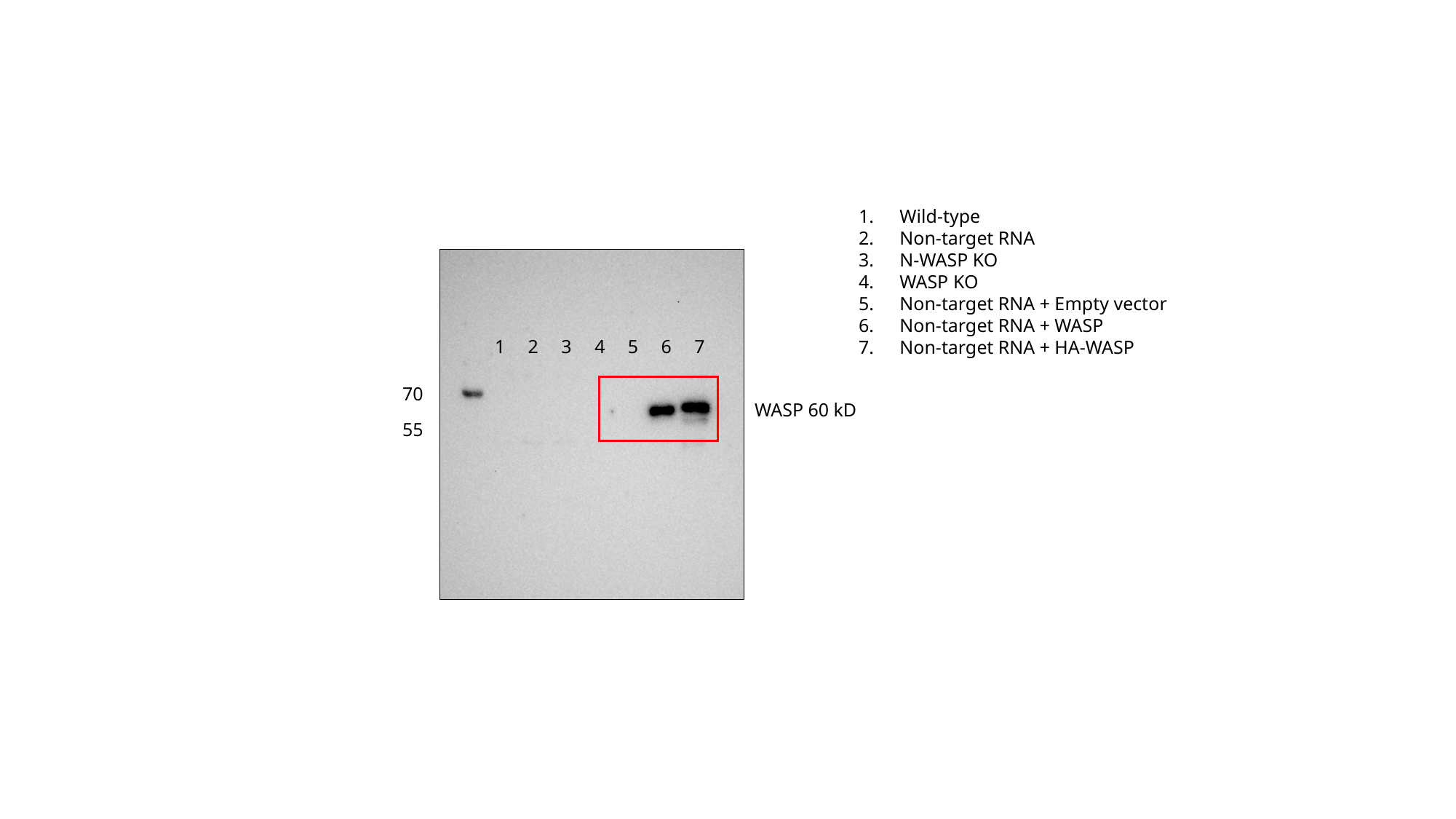

Wild-type
Non-target RNA
N-WASP KO
WASP KO
Non-target RNA + Empty vector
Non-target RNA + WASP
Non-target RNA + HA-WASP
1
2
3
4
5
6
7
70
WASP 60 kD
55

## Slide 2
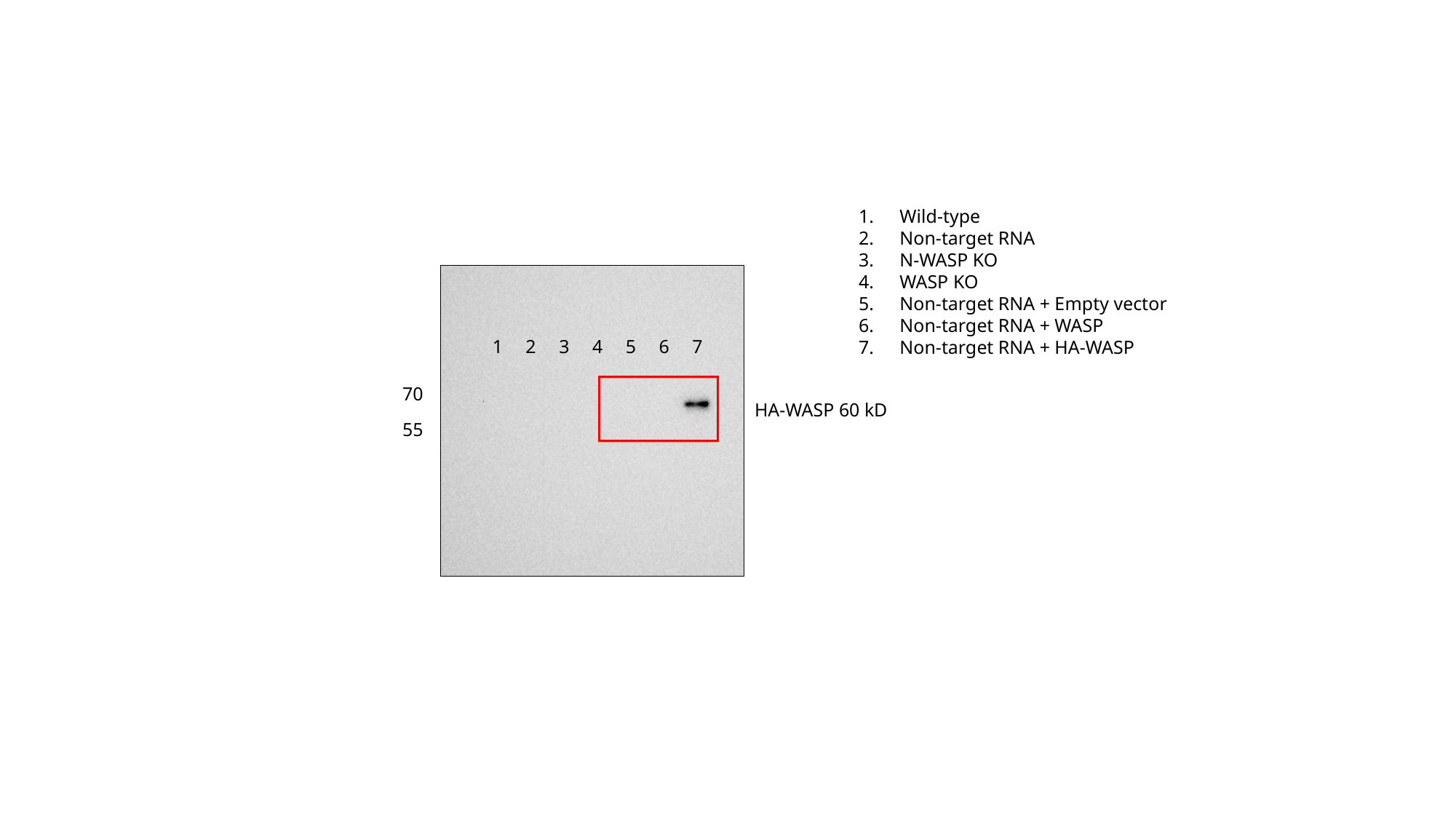

Wild-type
Non-target RNA
N-WASP KO
WASP KO
Non-target RNA + Empty vector
Non-target RNA + WASP
Non-target RNA + HA-WASP
1
2
3
4
5
6
7
70
HA-WASP 60 kD
55

## Slide 3
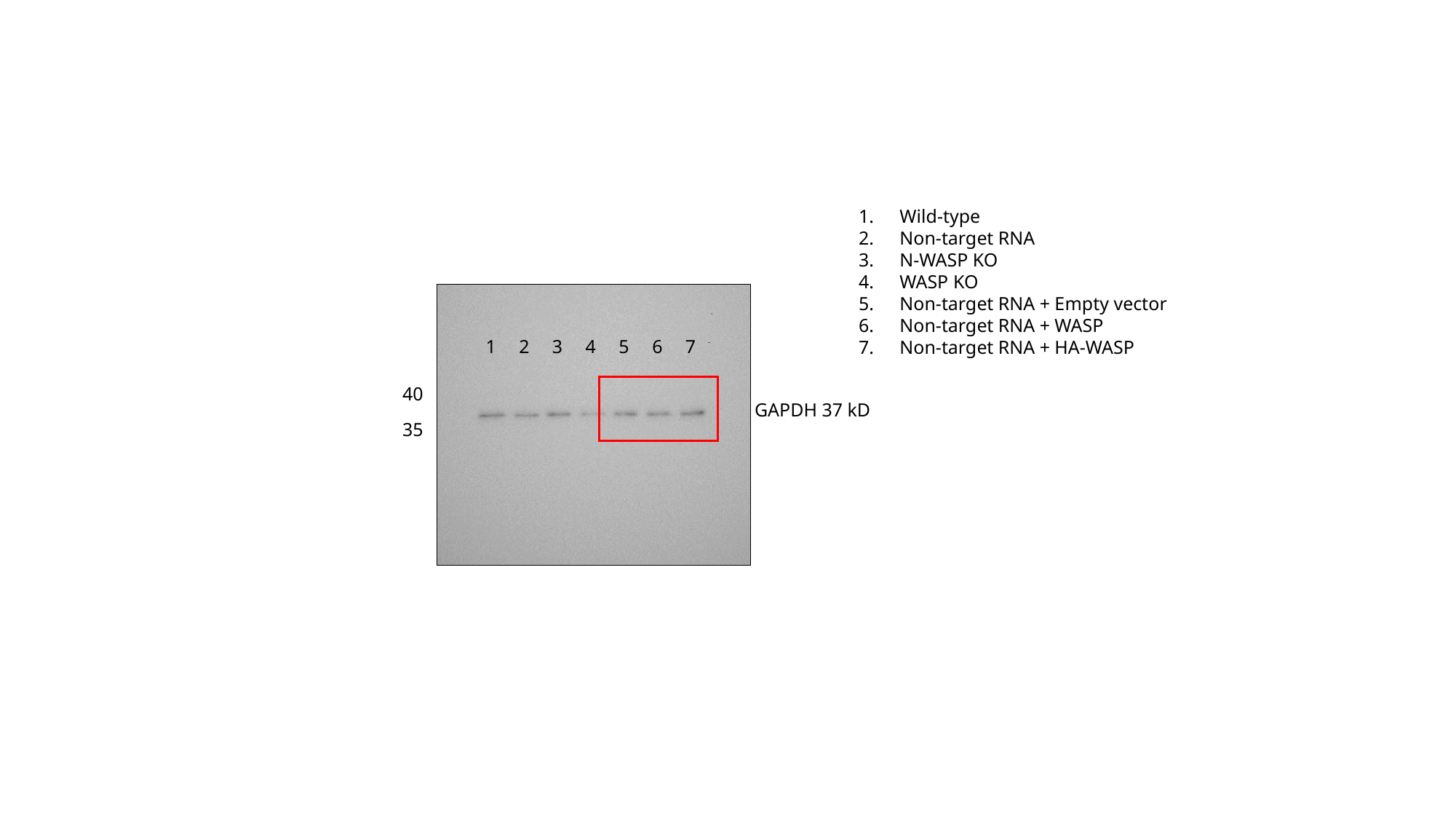

Wild-type
Non-target RNA
N-WASP KO
WASP KO
Non-target RNA + Empty vector
Non-target RNA + WASP
Non-target RNA + HA-WASP
1
2
3
4
5
6
7
40
GAPDH 37 kD
35
