# Supplementary figures and images for: Dispensable players: N-WASP and WASP are not crucial for homology-directed DNA repair
Source: EMBO Rep. 2026 Apr 10;27(10):2798–822. doi: 10.1038/s44319-026-00771-y (PMC13219447; doi:10.1038/s44319-026-00771-y)

## Slide 1
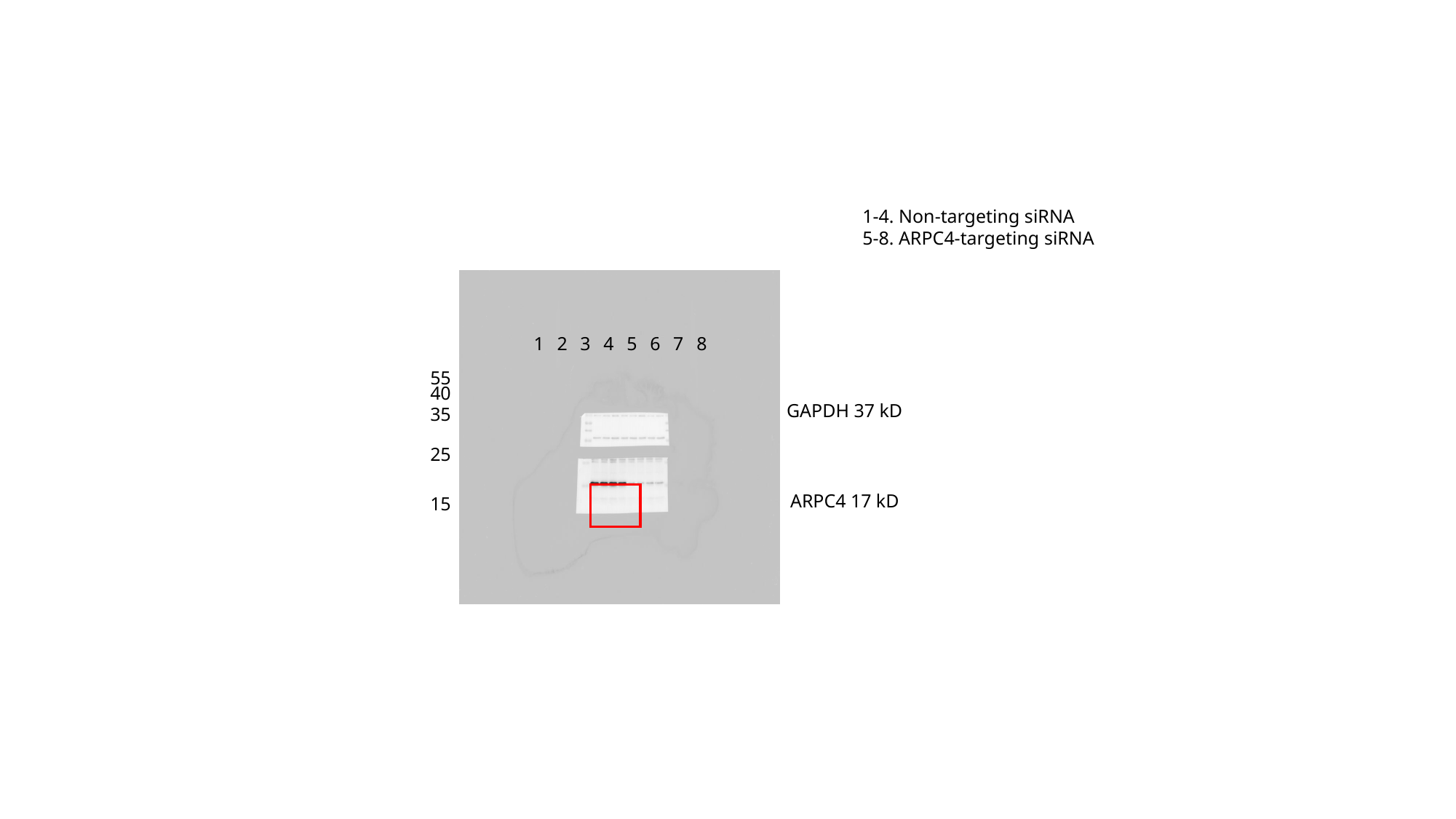

1-4. Non-targeting siRNA
5-8. ARPC4-targeting siRNA
1
2
3
4
5
6
7
8
55
40
GAPDH 37 kD
35
25
ARPC4 17 kD
15

Supplement: Supplementary file 8 — Source data Fig. 10 [file 44319_2026_771_MOESM8_ESM.zip › Figure 10/10C/Western Blot.pptx]
