# Supplementary material for: Dispensable players: N-WASP and WASP are not crucial for homology-directed DNA repair
Source: EMBO Rep. 2026 Apr 10;27(10):2798–822. doi: 10.1038/s44319-026-00771-y (PMC13219447; doi:10.1038/s44319-026-00771-y)
Supplement: Supplementary file 9 — Expanded View Figures [file 44319_2026_771_MOESM9_ESM.pdf]

Expanded View Figures

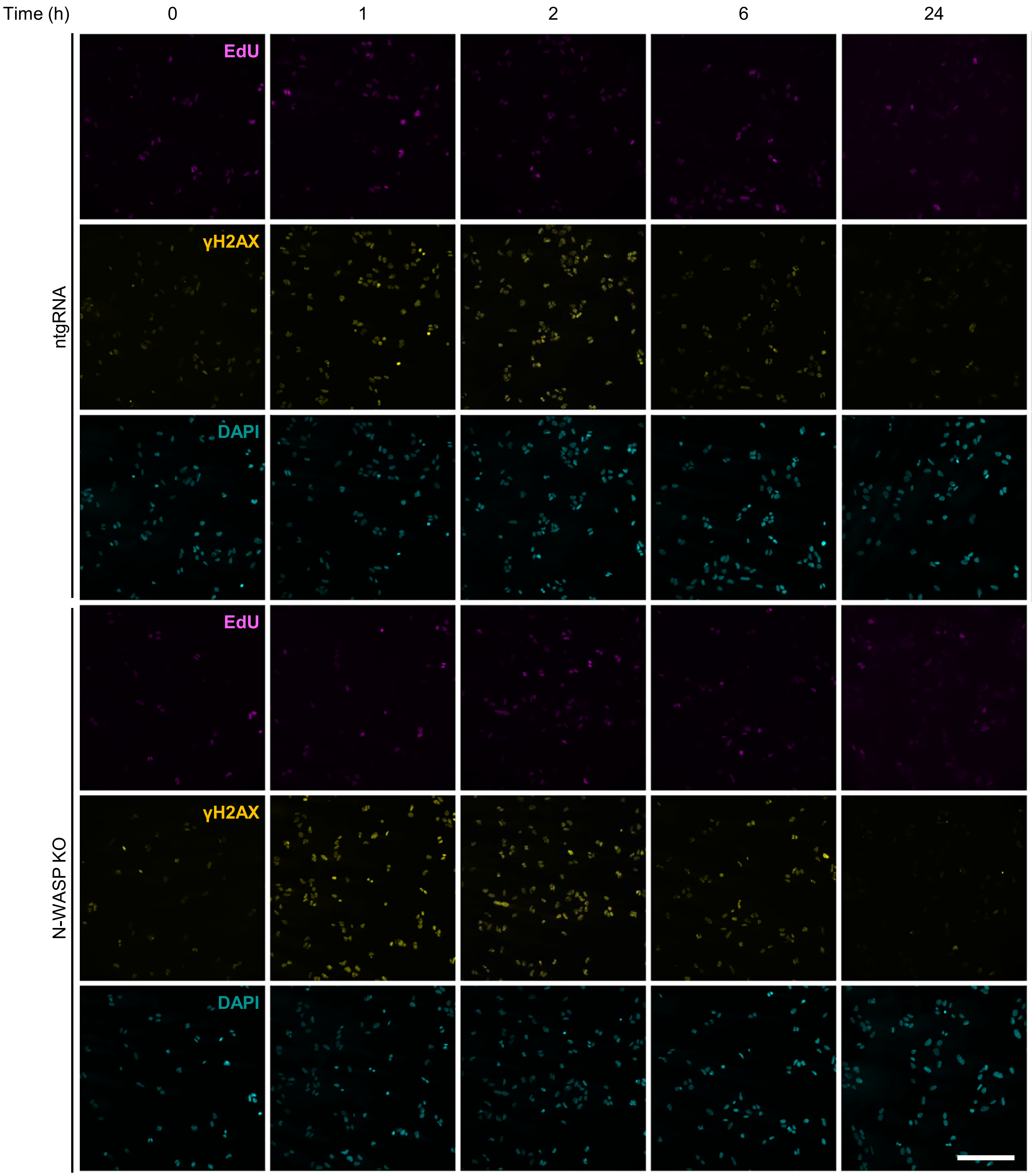

Figure EV1. Loss of N-WASP is not increasing gH2AX foci after irradiation.

Representative images of U2OS' γH2AX and EdU stainings at the indicated time points following exposure to 2 Gy X-ray irradiation; Sham-irradiated cells served as the 0 h controls. Cells were incubated with 10 μM EdU for 30 min prior to collection. Scale bar: 200 μM.

© The Author(s)

A

gRNA	ntgRNA		WASP KO	
Analysis tool	ICE	DECODR	ICE	DECODR
KO score	0	0	92	95.2

B

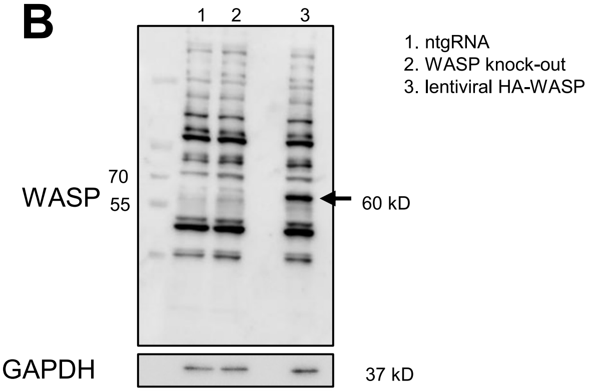

C

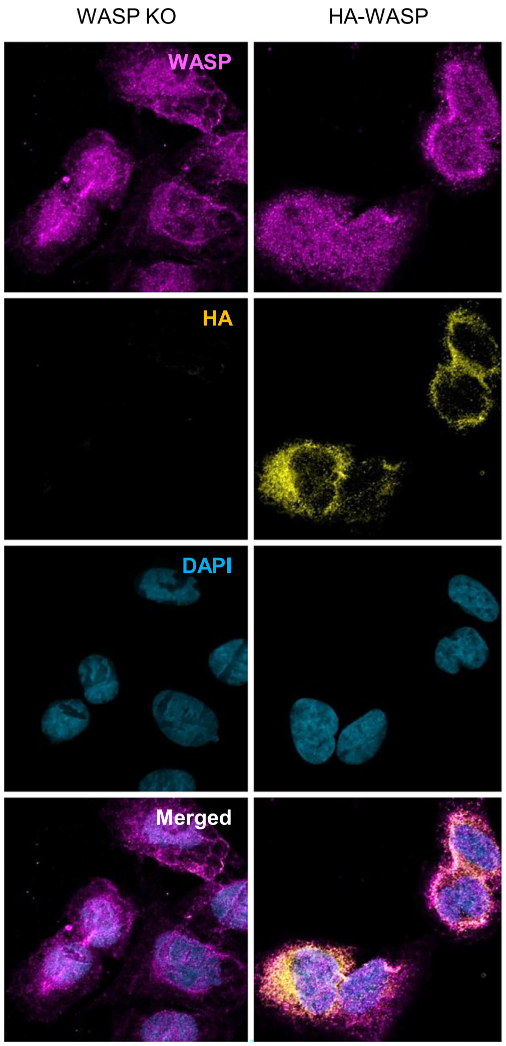

D

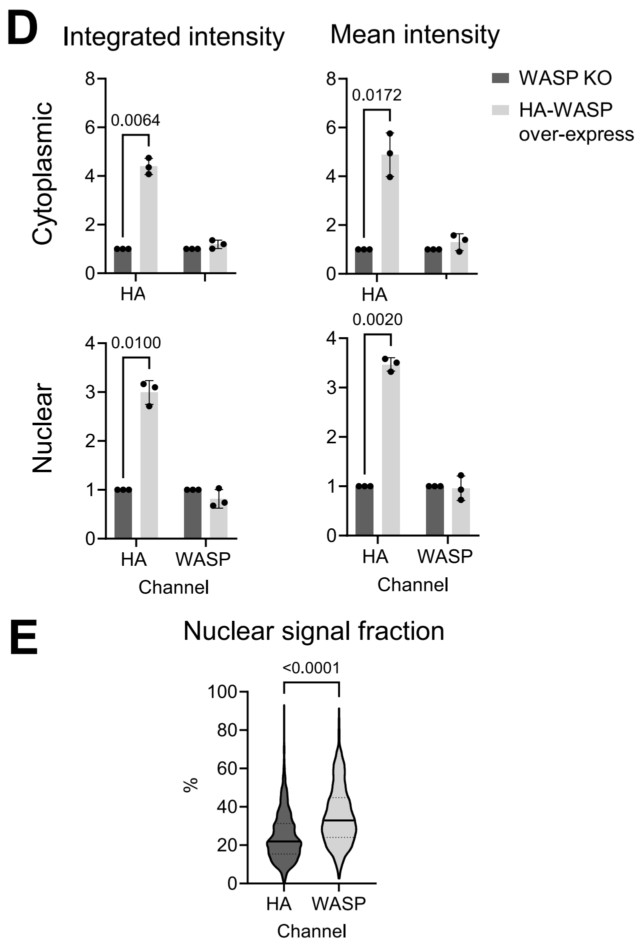

Figure EV2. WASP antibody validation.

(**A**) Assessment of WASP knock-out efficiency in U2OS cells through Sanger sequencing of genomic PCR products, followed by analysis using DECODR and ICE. (**B**) Immunoblotting of control transduced (ntgRNA), WASP knock-out, and HA-WASP-overexpressing (lentiviral HA-WASP) U2OS cell lines. (**C**) Immunofluorescence staining of WASP knock-out U2OS cells with or without overexpression of HA-tagged WASP via lentiviral vector. Scale bar: 20 μm. (**D**) Quantification of HA and WASP signal intensities in the two groups from (**C**), normalized to the WASP knock-out (W-KO) group. Each dot represents the average value from an independent biological replicate, containing 5 images for W-KO and 24–30 images for HA-WASP overexpression. Data are shown as mean ± SD, with statistical significance determined by a two-tailed unpaired *t* test. (**E**) Distribution of HA and WASP signal proportions in nuclear areas of images from the HA-WASP overexpression group, presented as a violin plot with median and interquartile range (lower and upper quartiles), statistical significance was determined by a two-tailed unpaired *t* test.

 © The Author(s)

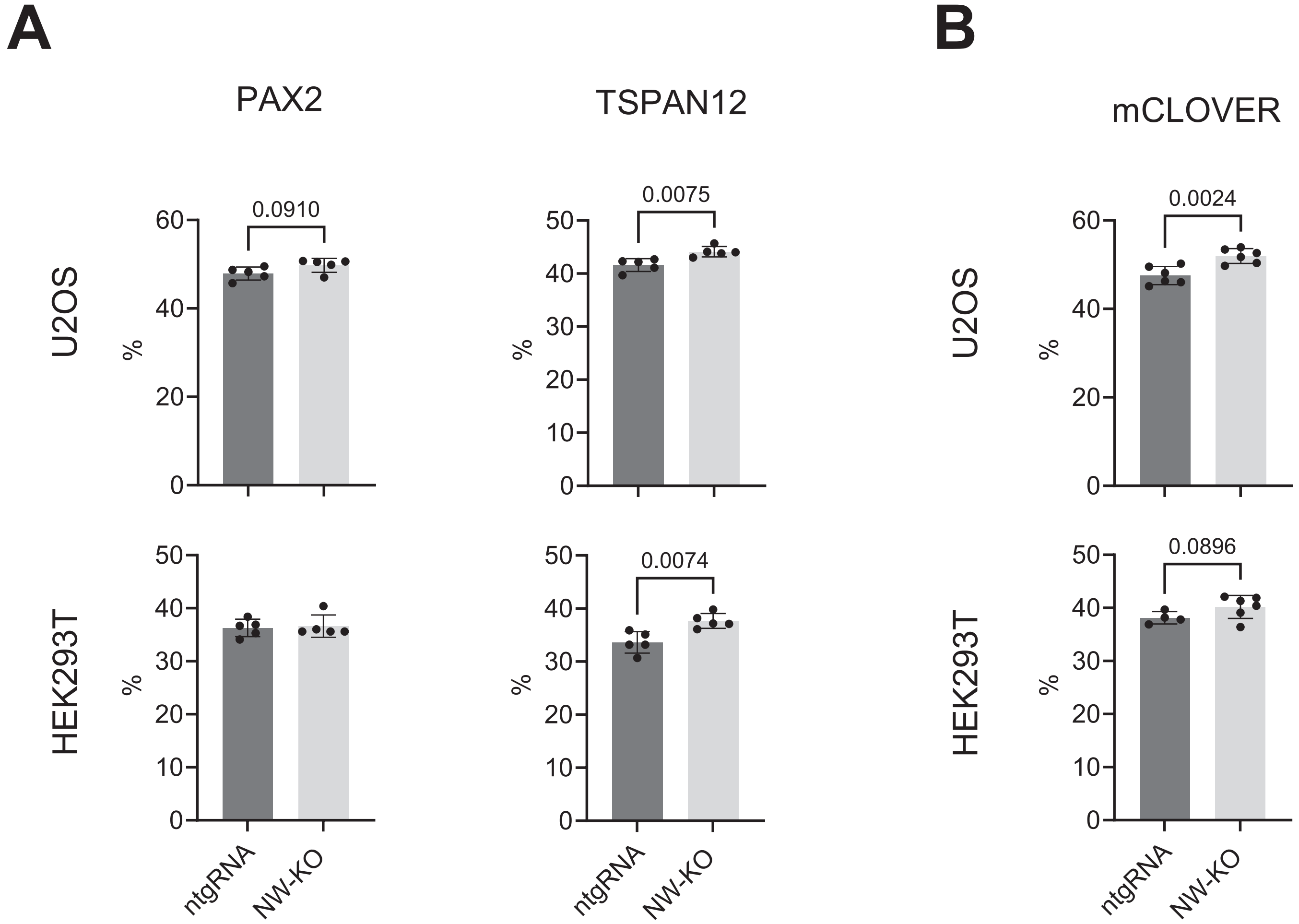

Figure EV3. The effects of N-WASP knockout on U2OS cell transfection.

Indicated cell types with (ntgRNA) and without N-WASP (NW-KO) were transfected with (**A**) plasmids encoding Cas9 and an sgRNA targeting indicated endogenous genes (TSPAN12, PAX2) and corresponding single-stranded repair templates for IDAA experiments, or with (**B**) Cas9, an sgRNA for LMNA, and a double-stranded repair plasmid inserting the fluorescent mClover. Transfection efficiency as determined by FACS for fluorescent transfection marker. Each dot represents an independent experiment. Values are shown as mean ± SD, with significance assessed by a two-tailed unpaired *t* test.

© The Author(s)
