# Supplementary material for: Dispensable players: N-WASP and WASP are not crucial for homology-directed DNA repair
Source: EMBO Rep. 2026 Apr 10;27(10):2798–822. doi: 10.1038/s44319-026-00771-y (PMC13219447; doi:10.1038/s44319-026-00771-y)
Supplement: Supplementary file 6 — Source data Fig. 7 [file 44319_2026_771_MOESM6_ESM.zip › Figure 7/7C/U2OS/ICE_Results_U2OS_ForwardPrimer.zip!/Understanding your ICE Export.pdf]

Understanding your ICE Export

Your ICE results will be maintained online for at least 6 months and can be accessed through your unique ICE results URL.

Additionally, your ICE results may be exported and downloaded as a zip file. The export file contains a summary file and a results subfolder for each sample/control pair included in your ICE analysis.

Summary file

The ICE export includes the summary data in two formats (Excel and .csv). The data included in both files are the same.

The summary file contains the following information (columns):

A. **Label**: This column contains the analysis label. If no label was provided, this column will include the name of the edited file.
B. **ICE**: This column includes the Indel Percentage. This includes all edits, regardless of whether they are expected to introduce a KO.
C. **KO-Score**: This column includes the KO Score, or all edits expected to introduce a KO (frameshift mutations or fragment deletions of 21 bp or greater).
D. **KI-Score**: For KI analyses only, this column will include the KI Score, or all sequences that contain the desired knock-in sequence in its entirety without any additional indels.
E. **ICE d**: This column contains the ICE-d Score. The ICE-d score reflects the discordance between the edited and control sequencing traces. Specifically, the ICE-d reflects the **Δ**d, or the difference between the control and edited alignment before the cut site and after the cut site. The ICE-d score typically lines up with the indel score. It is important to note that the ICE-d score is not well-suited to the analysis of a population edited using a multi-guide strategy.
F. **Mean Discord Before**: This column contains the Mean Discordance Score before the first cut site (a minimum score of 0 indicates no discordance between the control and edited sequences, a maximum score of 1 indicates high discordance).
G. **Mean Discord After**: This column contains the Mean Discordance Score after the first cut site.
H. **Guide Sequences**:This column contains guide sequences input into the ICE tool.
I. **Control Sample Quality Score:** Median Phred score (0-62) of the region before the first cut site in the control Sanger file, excluding initial noisy positions.
J. **Edit Sample Quality Score**: Median Phred score (0-62) of the region before the first cutsite in the edited Sanger file, excluding initial noisy positions.
K. **Notes**: If applicable, this column includes warning and error messages associated with the analysis.
L. **Indels**: This column summarizes the indels predicted to be present in the edited sample and the relative contribution of each indel (‘Indel size’: Contribution Percentage).

Results subfolders

For each paired analysis (control and edited sample files) included in your ICE results, your ICE export will include a subfolder with additional analysis-specific files.

File formats:

- **.json:** Easily importable by computer programs for further analysis.
- **.txt:** Formatted for visualization purposes.
- **.png:** Image format.
- **.svg:** Vectorized images for high-quality display and editing.

Files:

- **All:** Alignments using full sequences of control and edited samples.
- **Windowed:** Alignments using high-quality region of control sample before the first cut site, excluding initial noisy positions.
- **Contribs:** This file contains the sequences (contributions) predicted to be present in the edited sample and the relative contribution of each sequence.
- **Plot:** The file includes the Discordance and Indel Distribution plots captured in the ‘Indel Distribution’ tab of the online ICE results.
- **Trace:** The file contains a visualization of the alignments between the control and edited sequences across the first cut site.

Please note that your ICE export file will not include result subfolders for any analyses that could not be completed due to an error. For more information about specific error and warning messages, as well as troubleshooting recommendations, please refer to the Knockout and Knock-in ICE Analysis Protocols.
